# Surgeon’s imposter syndrome: a systematic review

**DOI:** 10.1007/s00423-024-03582-8

**Published:** 2025-01-18

**Authors:** Michael El Boghdady, Béatrice Ewalds-Kvist

**Affiliations:** 1 St George’s University Hospitals, London, UK; 2 University of Edinburgh, Scotland, UK; 3 Stockholm University, Stockholm, Sweden; 4 University of Turku, Turku, Finland

**Keywords:** Surgeon, Imposter syndrome, Impostor syndrome, Impostorism, Wellbeing

## Abstract

**Introduction:**

Imposter syndrome (IS) refers to the psychological experience of imagining that one’s achievements do not originate from one’s own authentic competence. Surgeons are constantly faced with life-threatening decisions and can easily feel inadequate or insecure despite their years of training and experience. Imposter syndrome can distress surgeons at all career stages and has profound psychological and professional consequences. We aimed to review imposter syndrome in surgeons.

**Methods:**

A systematic search was performed in compliance with The PRISMA checklist. Search was performed in the PubMed and ScienceDirect databases. We included articles about IS in surgeons. We excluded narrative articles, commentaries and studies involving medical students or other specialties. Citations were quality assessed by MERSQI and evidence graded (GRADE). Risk of bias was assessed among the included citations.

**Results:**

The search revealed 695 citations, from which a final list of 12 was compiled after applying the inclusion and exclusion criteria. Participants included trainees and consultant surgeons across various surgical specialties. The following research questions were answered: Are surgeons with IS predisposed to mental or physical challenges? Do surgeons experience gender differences in IS? Can the feeling of IS be reduced?

**Conclusion:**

There is a high prevalence of imposter syndrome among surgeons. Surgeons with IS are predisposed to experience mental or physical challenges. Female surgeons experience IS more frequently than their male counterparts. Feelings of IS can decline with increasing age but also with other included methods. Risks and multiple preventative measures were explored. The key to reducing IS is to train oneself to discern fact from fiction, thereby undermining distorted thoughts that perpetuate feelings of being an imposter.

## Introduction

Imposter syndrome (IS) or impostorism refers to the psychological experience of imagining that one’s achievements do not originate from own authentic competence, but from charm, being lucky, doing enormously overwork, or from having deceptively impressed other people [1]. IS is best described as multifactorial with demographic, familial, and environmental contributors [2, 3]. People with IS struggle with a form of inaccurate self-assessment that distresses those who cannot enjoy an internalized sense of success. IS is associated with perfectionism as well as is triggered by self-doubt and anxiety and is frequently observed in the critical culture of medicine [4; 5; 6].

Prevalence rates of IS varied from 9 to 82% largely depending on the screening tool and cutoff score used to assess symptoms which were particularly high among ethnic minority groups [7]. IS in medical students is often associated with depression, anxiety, and stress [8]. It is linked to impaired job performance and satisfaction, as well as compassion fatigue and dissatisfaction among various employee populations, including clinicians [9].

Surgeons are constantly faced with life-threatening decisions and can effortlessly feel inadequate or insecure despite their years of training and experience [10]. Surgeons’ stress originates from numerous factors such as the highly demanding work environment and is fostered by fear of failure as well as fear of success in their career development [11]. Imposter syndrome can affect surgeons at all career stages, leading to profound psychological and professional consequences. It is considered a systemic problem rather than a personal challenge. IS is frequently associated with burnout, suicidal ideation, compromised well-being, low self-esteem, and lower levels of professional satisfaction [12].

This study aimed to examine imposter syndrome in surgeons, addressing the following research questions:

A. Are surgeons with IS predisposed to experience mental or physical challenges?
B. Do surgeons experience gender differences in IS?
C. Can feelings of IS be reduced?

## Methods

### Data search

A systematic search was performed in compliance with The PRISMA (Preferred Reporting Items for Systematic Review and Meta-Analysis) checklist [13].

Search was performed in PubMed and ScienceDirect databases to April 2024. The search terms used were ‘*’ imposter syndrome AND surgeon’’*, ‘*’ impostor syndrome AND surgeon’*’, ‘’imposter syndrome AND surgery’’ and ‘*’impostor syndrome AND surgery’’*. The Medical Subject Headings (MeSH) terms used were ‘’impostor syndrome OR imposter phenomenon OR impostor phenomenon AND surgeon.

### Inclusion and exclusion criteria

We included citations about imposter syndrome with focus on surgeons. We excluded reviews, conference abstracts, semi structured interviews, letters to editors and citations that focused on IS in students or other professions.

A detailed literature search was performed, by inspecting titles, abstracts, and the full text of the relevant papers. The citations were reviewed against the inclusion and exclusion criteria. The inclusion and exclusion criteria were pre-determined, and the potential final citations were read in full text for further assessment of quality for analysis of the risk of bias across studies as well as for evidence grading.

### Quality assessment

The retrieved citations were available in full text. Quality assessments of the citations were performed using The Medical Education Research Study Quality Instrument (MERSQI) which contains 10 items that reflect 6 domains of study quality including design, sampling, type of data, validity, level of data analysis, and outcomes. For the assessment of the validity of evaluation instrument, we focused on face and/or external validity, limitations, and correlations with other instruments [14]. The MERSQI score represents the mean of two assessors’ quality estimations of each citation. Insufficient quality of a citation had < = 12,25 scores, low quality was between 12,26 − 12,63 scores, moderate quality was placed between 12,64 − 12,88 scores and high quality started from 12,89 + scores.

### Evidence grading

Evidential value for the use of evidence grading was based on criteria for employing GRADE, which comprised four grades [15]:

- Evidence grade I: strong scientific evidence based on at least 2 studies with high evidential value or a systematic review/meta-analysis with high evidential value.
- Evidence grade II: moderate scientific basis: a study with high evidential value and at least 2 studies with moderate evidential value.
- Evidence grade III: low scientific evidence: a study with high evidential value or at least 2 studies with moderate evidence value.
- Evidence grade IV: insufficient scientific evidence: 1 study with moderate evidence and/or at least 2 studies with low evidential value.

### Analysis

Data were extracted and analysed using IBM SPSS version 26 and Microsoft Excel. Information collected for each citation included citation’s description (title, authors, year of publication, number of surgeons), aim, findings, MERSQI scores and evidence grade.

## Results

**Fig.1:**
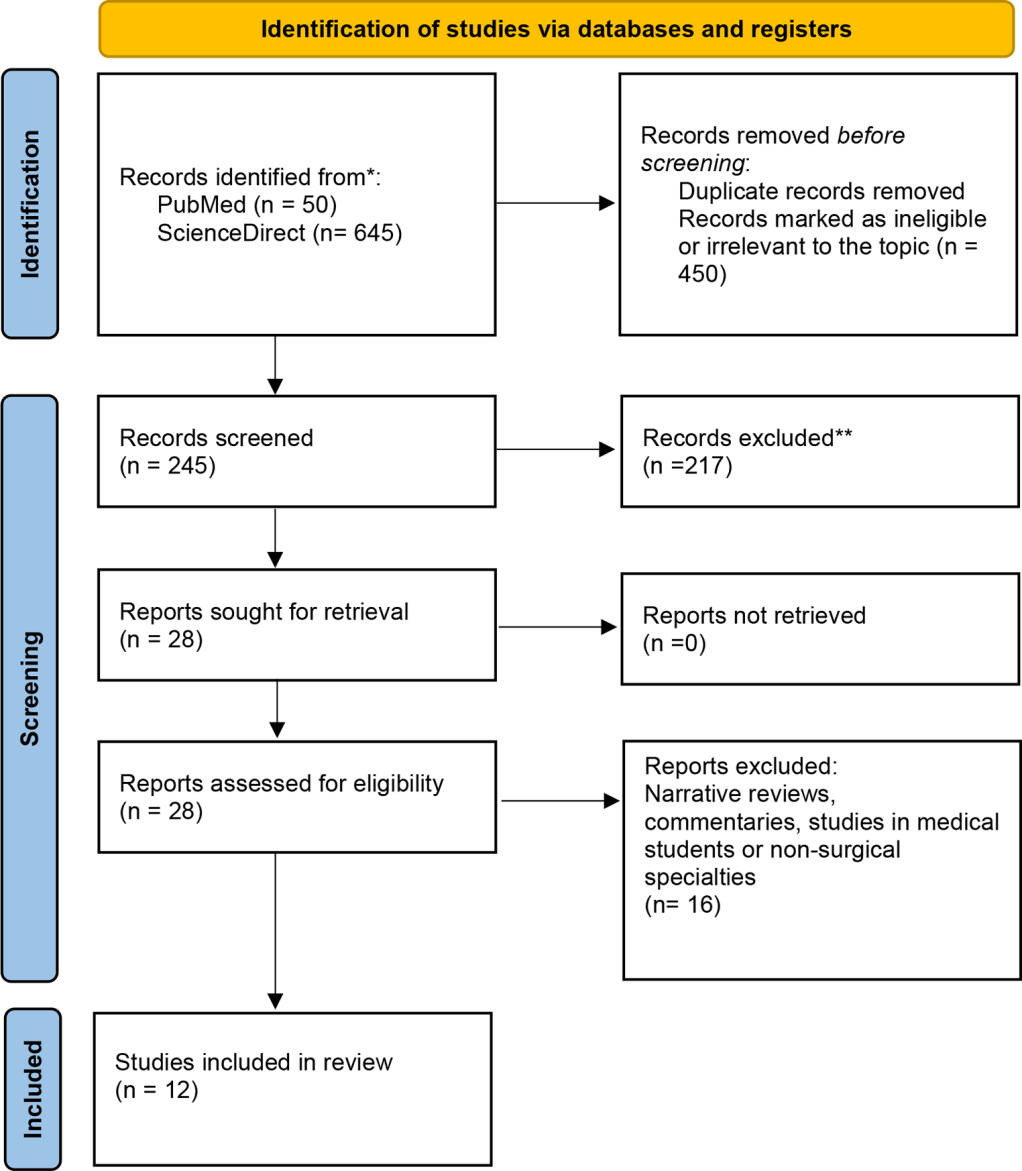
The PRISMA flow diagram for the systematic search

**Fig. 2:**
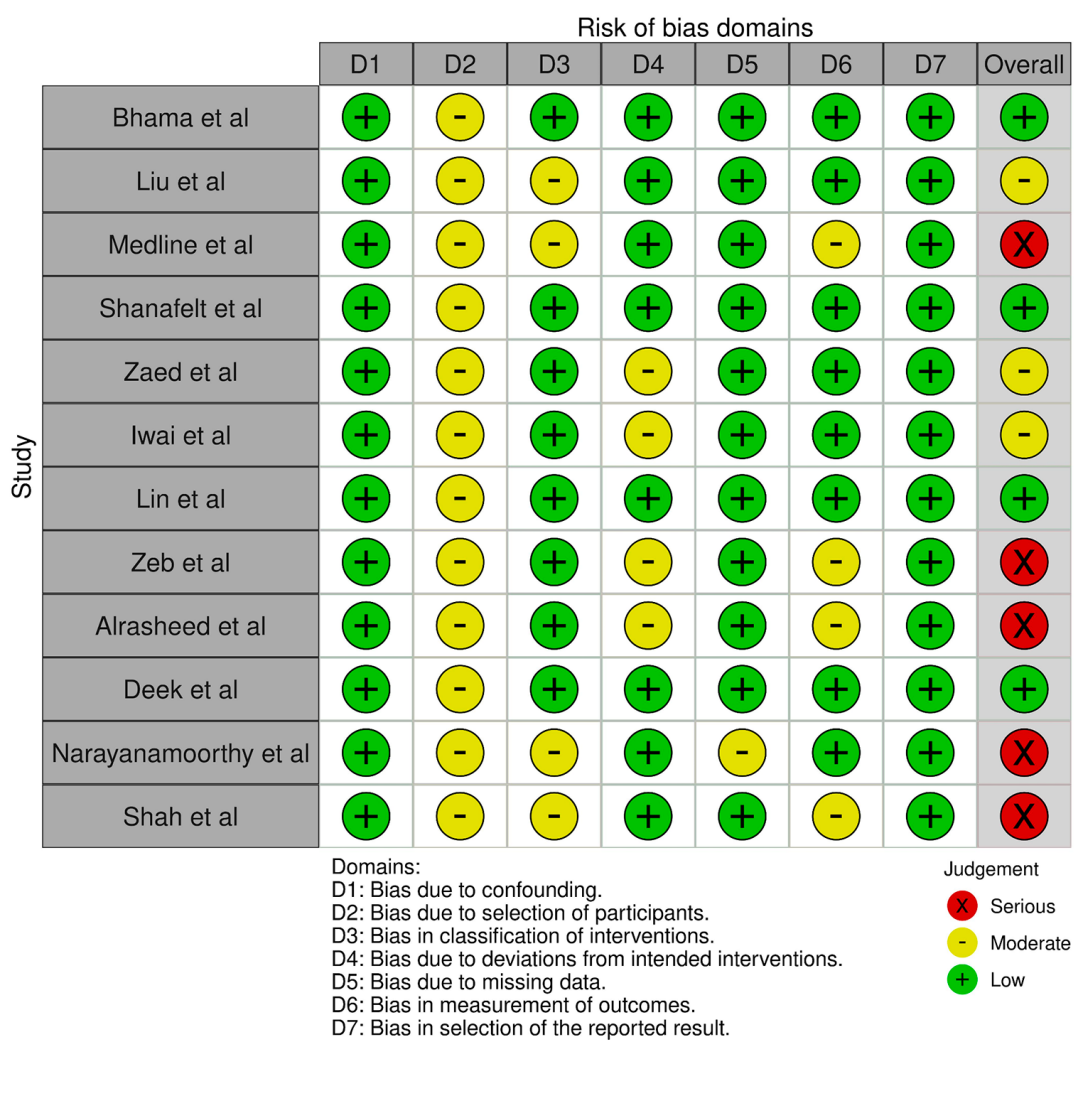
Incorporated studies evaluated against the Risk of Bias tool

**Fig. 3:**
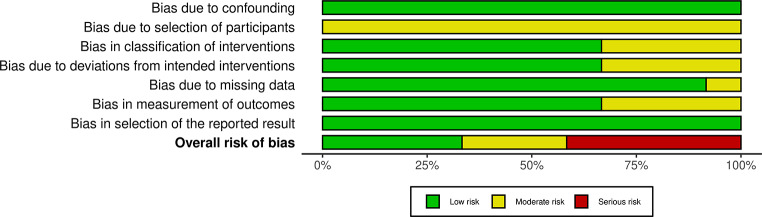
Summary of Risk of Bias across studies

The search revealed 695 citations, from which a final list of 12 citations was compiled after applying the inclusion and exclusion criteria (Fig 1) (Table 1) [16–27]. Participants included trainees and consultant surgeons from various surgical specialties such as general surgery, neurosurgery, ophthalmology, orthopaedic surgery, otolaryngologists, oral and maxillofacial surgery and urology. All included studies were cross-sectional and analysed for risk of bias (Figs. 2, 3). The citations were quality assessed by means of MERSQI and yielded scores as follows: Insufficient quality (*n* = 4) *= 12.25;* Low quality (*n* = 2) = 12.26–12.63; Moderate quality (*n* = 3) 12.64–12.88 and High quality (*n* = 3) *≥* 12,89+ (Table 1). The risk factors, prevalence and preventative measures for imposter syndrome were analysed (Table 2).

**Table 1:** Tabular analysis of the included citations

Author	Aim of the study	Subjects	Findings	MERSQIscore
Bhama et al. [16]	To study the level of IS in general surgery trainees. The Clance Impostor Phenomenon Scale was administered.	n = 144 trainees The largest age group was 29-30 yr. Male = 68 Female = 75 Other = 1	Only 22.9% had “none to mild” or “moderate” imposter syndrome (IS). A majority (76%) had “significant (frequent)” or “severe” IS. There was no difference between junior to senior trainees. There was no gender difference.	
Liu et al. [17]	To investigate the relationship of anxiety and impostorism (CIPS) to burnout in postgraduate medical learners	n= 269 trainees Family medicine = 29.4% Pediatric medicine = 31.6% Anaesthesia = 20.5% General Surgery = 21.6% Median age group = 25-34 yr. (89.6%) Male = 105 Female = 164	IS was identified in 62.7% of all participants. The average score on the Clance Impostor Phenomenon Scale (CIPS) was 66.4 (SD = 14.4), corresponding to recurrent feelings of IS. Female were at higher risk for IS.	Moderate12.75
Medline et al. [18]	To rate the following personality traits: self-efficacy, IS, assertiveness, perfectionism, and self-rated likeability.	n = 296 (different surgical sub-specialties) 161 Consultant surgeons 135 various stages of surgical training Median age group = 25-40 yr. Female = 161 Male = 135	Surgeons are a self-efficacious group of perfectionists with widespread variability in IS and assertiveness. Female gender and younger age were associated with more IS and less assertiveness, highlighting an opportunity for early career coaching.	
Shanafelt et al. [19]	To study the prevalence of (IP) among physicians and assess its link to skill characteristics, professional fulfillment, burnout, and suicidal ideation	n = 3116 (105 General Surgery, 241 surgery subspecialty, 28 neurosurgery, 135 ophthalmology, 168 orthopedic surgery, 17 urology) Male = 1934 female = 1185 Other = 3 Missing = 10 Median age group = 55-64 yr.	Between 4% (133) and 10% (308) each of the 4 IS items as a “very true” characterization of their experience. Relative to those with a low IS score, the odds ratio for burnout among those with moderate, frequent, and intense IS was 1.28, 1.79, and 2.13, respectively.A similar association between IS and suicidal ideation was observed.On multivariable analysis, physicians had a greater intensity of IP than workers in other fields in response to the item. Females have more IS experience than male surgeons.IS experiences are associated with increased burnout and suicidal ideation and lower professional fulfillment	High13.75
Zaed et al. [20]	To study the prevalence and severity of imposter syndrome (IS) by means of ‘Clance Imposter Phenomenon Survey (CIPS).	n = 103 trainees in neurosurgery Subspecialties = 8 Median subspecialty group = neurosurgery Oncological surgery = 39 Male = 59 Female = 44 Median age group= 26-30 yr.	The prevalence rate of IS was 81.6%. Among the respondents with IS, 42.7% showed moderate signs, 27.2% frequent, and 11.7% had an intense IS symptomatology. Education, female gender, and academic achievements were identified as predictive factors of IS.	
Iwai et al. [21]	To study leadership and Imposter Syndrome in surgeons	Consultants and retired physicians = 2,183 Surgical specialty = 464 In leadership positions = 345 1,471 (67.4%) were in leadership roles and 712 (32.6%) were not Cisgender female/woman = 1148 (52.6 %) Cisgender male/man = 987 (45.2%) Transgender/nonbinary/other = 15 (0.7%) Age, median = 48 yr. (39–60)	Female surgeons described more often IS compared to male surgeons, even when female surgeons had taken leadership roles. Similar trends were observed for female and male non-surgeons. IS rates did not differ between race and ethnicity, including among those underrepresented in medicine, even after adjustment for gender and leadership role.	Moderate12.75
Lin et al. [22]	To study if intolerance of uncertainty and confidence in problem-solving skills are independently related with feelings of imposter syndrome after accounting for other factors	Orthopedic surgeons specializing in upper extremity = 102 Men = 91 Women = 11 Mean age = 52 yr.	Greater feelings of IS were moderately to strongly associated with greater intolerance of uncertainty and lower confidence with problem solving. This finding helps to confirm that feelings of IS may relate as much to one’s inner narrative as to one’s circumstances; both approaches for mindset training (exercises for an ideal inner narrative) combined with peer surgical coaching programs that foster both a growth mindset as well as a culture of inclusion, belonging, and mutual support merit study for the ability to help limit surgeons’ feelings of IS and improve joy in practice.	High﻿13.00
Zeb et al. [23]	To assess the prevalence of imposter syndrome amongsurgical trainees	Surgical trainees = 156 Men = 104 (66.7%) Women = 52 (33.3%)	Moderate impostorism was seen in 81(51.9%) of the respondents and 57 (36.5%) respondents reported severe or intense IS.Among postgraduate trainees, no significant differences in Clance Imposter Phenomenon Scale score by year was noted (n.s.).IS was highly prevalent among surgical trainees, i.e. 138 (88.5%) falling in the range of either moderate, severe, or intense impostorism.	
Alrasheed et al. [24]	To determine the prevalence of imposter syndrome (IS) among otolaryngologists practicing in Saudi Arabia.	Otolaryngologists = 80 Residents/trainees = 46 (57.5%) Fellows = 10 (12.5%) Registrars = 9 (11.3%) Consultants = 15 (18.8%) Males n = 46 (57.5%) Females n = 34 (42.5%)	A total of 27.5% prevalence rate of IS among otolaryngologists was found with a mean IS of 56.79 ± 12.98. In terms of severity, 62.5% (n = 50) had a moderate level of IS, 25 % (n = 20) had high IS, and 5 % (n = 4) had intense IS.IS was more common in trainee otolaryngologists as compared to consultants, but there was no association in terms of gender, type of hospital, or fellowship subspecialty. Trainees were more susceptible than consultants and fellows.	
Deek et al. [25]	To evaluate the frequency and severity of Imposter Syndrome (IS) in in oral and maxillofacial surgery (OMS) trainees as well as to identify factors associated with higher Clance Imposter Phenomenon Survey (CIPS) scores.	Trainees = 175 Male = 72.6% Female = 25.7% Preferring not to say = 1.7% The average age of respondents = 31 yr.	The average CIPS score was Md 59.8. There was relatively even distribution between categorical level of training of respondents.Eleven male respondents (6.29%) had few IS scores <40; 84 (45.0%) of respondents had moderate IS (score >40 - <61); 71 (40.6%) had frequent IS and 9 including 5 females (5.1%) suffered from intense IS experiences (score >80). The average male trainee experienced moderate IS, while the average female trainee experienced frequent IS.	Moderate12.75
Narayanamoorthy et al. [26]	To analyse the female prevalence of IS in different surgical specialties in US, associated risk factors, and to determine the association between IS and mental conditions like anxiety and depression.	Trainees = 382 Otolaryngologists = 46 General surgery = 75 Orthopaedics = 31 Opthalmology = 28 Urology = 31 Men = 118 (30.9%) Female = 264 (69.1%) Age = 25-35 yr.	The prevalence of IS was higher among female trainees. Risk factors comprised being single, having no dependents (children), being a foreign graduate and having feelings of anxiety. Female trainees belonging to surgical fields with heavy workload, low confidence in skills, low work satisfaction and long working hours risk to be affected by IS.	
Shah et al. [27]	To study burnout and self-compassion, imposter syndrome, moral injury, flourishing and trauma symptoms by discrimination scale between non-surgical and surgical trainees.	186/1017 were compared based on postgraduate year of training and by specialty: surgical (186) versus nonsurgical specialty Most participants cisgender = 97% Transgender = 2.7% Male = 1.15% Female = 97% Mean age 31 yr. (29– 33)	Physician burnout is highly prevalent in the United States and is disproportionately experienced by physician trainees and women physicians.Self-compassion, IS, moral injury, flourishing and trauma symptoms recorded by discrimination scale did not differ between non-surgical and surgical trainees. IS and burnout affect medical and surgical trainees in a similar way.	High13.00

**Table 2:** Risk factors, prevalence and preventative measures for imposter syndrome (IS)

Author	Risk factors and prevalence for IS	Management and preventative factors for IS
Bhama et al. [16]	Prevalence of IS =76% (frequent or severe) in general surgery trainees. There is something either inherent to those choosing general surgery or the general surgery training culture that leads to such substantive levels of IS.	Identifying when IS change from a healthy protective mechanism to a negative energy leading to burnout and mental illness. Examining this change could help identify points of intervention to prevent and treat burnout in the general surgery trainee population.
Liu et al. [17]	The prevalence of IS was 62.7%, independent of specialty, and it contributes to anxiety and burnout. IS was identified as an independent risk factor for both anxiety, with a threefold increase, and burnout, with a twofold increase compared to baseline levels	Initiatives to mitigate impostorism may improve postgraduate learners’ wellness by reducing anxiety and burnout.
Medline et al. [18]	Female surgeons experience higher degrees of burnout and IS compared with their male counterparts; it is imperative that this gender gap is addressed. Although standardizing operative and clinical understandings may help develop confidence in all surgical trainees, greater attention should be given to younger female trainees to identify who may benefit from supplemental mentorship, both within and outside of the operating room.	With appropriate resources (supplemental mentorship, both within and outside of the operating room), students, and females in particular, could be empowered to take accountability for their own personal growth, fostering a more nurturing and proactive surgical culture.
Shanafelt et al. [19]	Aggregate IS scores by linear regression analysis were increased among women physicians. The IS scores varied by specialty. IS was independently associated with risk of burnout after adjusting for age, gender, relationship status, specialty, hours worked per week, practice setting, and self-valuation. IS was also independently associated with low professional fulfillment after adjusting for the same factors. IS was also independently associated with suicidal ideation.	Intentional efforts to mitigate IS by debunking collective attitudes that see physicians as superhuman with above basic human needs and stigmatize help-seeking as weakness. Such attitudes can be replaced with a culture of authenticity and vulnerability. Efforts to instill a growth mindset during the training process, reduce the stigma associated with help-seeking, and create a culture of vulnerability with colleagues are critical. By normalizing help seeking and reducing stigma, mindsets of perfectionism and “unforgiving excellence” culture changes to a culture of excellence and growth instead.
Zaed et al. [20]	IS was prevalent in young neurosurgeons (81.6%). Education, female gender and academic achievements were identified as riskfactors. In the multivariate analysis a correlation was found between an intense level of IS and female gender, the pursuit of an academic career, or working in an academic hospital. In the multivariate analysis, a correlation was found between female gender and the severity of the IS, the number of hours worked per week, and the number of articles read as well as between having a PhD degree and IS.	The academic environments may inherently scale down the self-perceptions of trainees. Perhaps being part devoted to the perpetual progress of medical science might turn out to be an incessant reminder of what is yet to be known, discovered, or improved.
Iwai et al. [21]	Female surgeons were more likely to report IS compared to male surgeons. Similar trends were appreciated for female and male non-surgeons, where female non-surgeons had higher rates of IS than their male counterparts. Female surgeons had higher rates of IS compared to male surgeons in leadership roles (81.5%). Females experience more bias, mistreatment, and workplace harassment, which may cause them to internally call into question their place of holding a leadership role.	Continuous professional development through peer mentoring and video-based surgical coaching programs show promise for providing accessible support to faculty at various levels of training and in diverse geographic settings.
Lin et al. [22]	Greater feelings of IS were modestly to strongly associated with greater intolerance of uncertainty and lower confidence with problem solving.	Strategies to alleviate discomfort with uncertainty and increase confidence in problem solving have the potential to help surgeons decrease feelings of IS and bolster joy in practice. Both cognitive behavioral therapy for mindset training (exercises for an optimal inner narrative) combined with peer surgical coaching programs that foster both a growth mindset as well as a culture of inclusion, belonging, and mutual support merit study for their ability to help limit surgeons’ feelings of IS and improve joy in practice.
Zeb et al. [23]	Among general surgery trainees the prevalence of moderate to significant, and intense IS was 88.5% and mild to none was 11.5%. Female trainees were more affected than male trainees. The year of training did not have any impact on the grade of IS.	Strategies need to be invented in institutions and at the national level to overcome the degree of IS among trainees. This will help the students to recognize and utilize their skills which will have a positive influence on their health and lead to improvement in the quality of care in hospitals.
Alrasheed et al. [24]	The prevalence of IS among otolaryngologists in Saudi Arabia was 27.5%. A total 42.5% had moderate IS, 21.3% had severe, and 6.2% had intense IS. Medical students and trainees with low self-esteem report elevated levels of IS. Perfectionistic tendencies have also been identified as a contributing factor to an increased risk of IS.	Early interventions aimed at identifying and managing IS during medical training may have the most impact. It is important to develop standardized interventions in healthcare and educational settings. A more nuanced, cross-cultural understanding of IS, allowing for more effective, culturally sensitive interventions is needed. The relationship between IS and the level of training suggests that career progression may alleviate IS symptoms, offering a pathway for potential interventions that support career development and mentorship as a means of reducing IS.
Deek et al. [25]	Oral and Maxillofacial Surgery trainees had a mean Clance Imposter Phenomenon Survey (CIPS) of 59.8, placing the average trainee on the higher end of the moderate imposter experience (IE) category. Female trainees were found to have a mean CIPS of 66.9 vs. male counterparts 56.5. IS is reportedly associated with low self-esteem, burnout, depression, anxiety, and psychological distress. It has also been linked to lower confidence in problem-solving and intolerability of uncertainty, as well as lower enjoyment in day-to-day practice.	Identifying the IS early may permit adaptive coping strategies to be implemented while the individual is still in training. Academic mentorship, normalizing IS feelings, and recognition of achievements thus far might help to reduce the psychological burden and allow those experiencing IS to minimize it.
Narayanamoorthy et al. [26]	A higher prevalence of frequent and intense IS, among female surgical trainees was found. Risk factors were being single, having no dependents, and being a foreign medical graduate. Feelings of anxiety correlated with IS. Surgeons are ambitious perfectionists who are expected to have assertiveness and self-efficacy. This self-imposed pressure can lead to IS and anxiety.	Wellness programs in training should educate all genders, to grow their inherent abilities. Programs should encourage their trainees to join medical associations to pair them with senior role models and mentors to advance leadership skills, confidence and strategies to achieve their goals. Programs should also take steps to generate a culture more acceptable to trainees from various international backgrounds.
Shah et al. [27]	Burnout was prevalent among all surveyed trainees with 63% scoring positive. Trainees also scored high in IS and moral injury with low levels of self-compassion, although respondents also reported themselves flourishing. Several risk factors have been identified that account for gender differences (females are more vulnerable), including unequal patient expectations, role expectations outside of work, and personal experiences within the workplace.	Targeted interventions for well-being, such as coaching, can help decrease the levels of burnout experienced by female physician trainees and do not need to be specialty specific

**Table 3:** Evidence-based answers to the research questions

Evidence grade II	**Are surgeons with IS predisposed to mental or physical challenges?**
High Moderate Moderate	**Shanafelt et al.**: IS experiences are associated with increased burnout in forms of emotional exhaustion and depersonalization of burnout and suicidal ideation. **Liu et al.**: IS was an independent risk factor with a threefold greater risk for anxiety and approximately twofold greater risk for burnout compared to the general population. **Iwai et al.**: The few females who attain leadership roles face barriers that amplify feelings of IS, regardless how high they climb. Female physicians promoted in academic medicine often face malicious behaviour from male colleagues, witness men with lesser achievements advance, and receive explicit messages suggesting that tenure decisions were related to their gender.
Evidence grade II:	**Do surgeons experience gender differences in IS?**
High Moderate Moderate	**Shanafelt et al.**: The IS experiences were significantly more severe among female physicians. *p*<0.001. IS had significant association to occupational distress and correlated with professional dissatisfaction. **Deek et al.**: From univariable analysis, male trainees were found to have a lower average CIPS scores compared with female respondents overall (*P* < 0.001) and female trainees were significantly more likely to have intense IS experiences (*P* < 0.001) **Iwai et al.**: Female surgeons described more often IS compared to male surgeons (90.0% vs. 67.7%; *p* < 0.001), even when female surgeons had taken leadership roles. Similar trends were observed for female and male non-surgeons.
Evidence grade I:	**Can feelings of IS be reduced?**
High High	**Lin et al.**: Feelings of IS may relate as much to one’s inner narrative as to one’s external circumstances. Strategies aime reducing uncertainty and increasing confidence in problem solving can alleviate feelings of IS and enhance joy in practice. Both *cognitive behavioral therapy* for mindset training (exercises for an optimal inner narrative) combined with peer surgical coaching programs that foster both a growth mindset as well as a culture of inclusion and belonging help limit surgeons’ feelings of IS. **Shanafelt et al.**: Feelings of IS relate to collective attitudes that see physicians as superhuman with superhuman needs. To stigmatize help-seeking as weakness needs to be replaced. By *normalizing* help seeking and reduction of the mindsets of a culture of perfectionism replaced by a culture of fostering a growth mindset with inclusion and belonging, limit surgeons’ feelings of IS.

The adjusted ratings gave a Spearman rho of 0.833, *p* < 0.001. MERSQI produces a potential range from 5 to 18 scores and presently the scores ranged from 9 to 14. The maximum scores for each domain were 3. The mean quality score for the included citations was computed to be 12,42 (SD = 1,02) scores. By means of visual binning the quality scores were divided with 25% of citations in each range.

The IS scores revealed were originated from Clance Imposter Phenomenon Scale (CIPS). The total score ranges from 20 to 100, with higher scores indicating a stronger presence of impostor feelings. As regards IS scores < 40 denotes few symptoms; scores > 41 to < 60 = moderate IS experiences; a score > 61 to < 80 means frequent IS feelings; IS scores > 80 implies intense IS experiences. The higher the score, the more frequently and seriously the IS restricts an individual’s being [19].

### Answer to research questions

Our three research questions were answered based on quality assessed evidence-based citations:

A. Are surgeons with IS predisposed to mental or physical challenges?
B. Do surgeons experience gender differences in IS?
C. Can feelings of IS be reduced?

The answers to questions A and B were assessed as moderate quality and consequently comprising evidence grade II.

The answer to question C was evaluated to possess high quality and subsequently holding strong evidence-based grade I. (Table 3).

## Discussion

### Are surgeons with IS predisposed to mental or physical challenges?

The surgeon’s anxiety, stemming from an achievement-related task, is often due to a fear of failure, which can lead to feelings of shame. For surgeons, both success and failure, as well as dealing with death, are inherent aspects of their profession. Surgeons with IS internalize failure but attribute success to external factors and create reasons why they do not deserve praise or credit. The fear of success shields the surgeon in his distorted inner dialogue from the idea that success will generate higher expectations from others and he will inevitably not be able to meet these expectations [28]. IS often leads to procrastination and overpreparation, as tasks that one is objectively capable of performing triggers the uncomfortable feelings of stress and fraudulence. However, these behaviors do not originate from laziness but rather represent efforts to alleviate the negative emotions generated by the task [29].

Imposter syndrome is not recognized as an official mental-health diagnosis neither in Diagnostic and Statistical Manual of Mental Disorders [10] nor in International Classification of Diseases [7, 10]; however, mental-health professionals recognize it as a straightforward and specific form of intellectual self-doubt. Yet, there is a strong association between physicians’ IS scores and mental distress in forms of burnout and suicidal ideation [10].

### Do surgeons experience gender differences in IS?

Previous research found that female plastic surgery trainees and faculty have a higher overall incidence of IS compared with males, and that female gender was the only independent factor associated with higher impostor scores after multivariable adjustment [30]. This gender difference agrees with results from our study where female gender is a significant risk factor for IS. Throughout their careers, females in medicine might be at higher risk of facing discrimination and microaggressions from patients, colleagues, supervisors, and staff, which hampers their career progression to higher positions [28]. A recent review of 33 studies reported that women exhibited statistically significantly higher rates of impostor feelings than men, while 17 studies found no difference between genders. This review demonstrated that while IS is common in women, it also affects men [7].

### Can feelings of IS be reduced?

Impostor phenomenon leads to coping through avoidance. However, active coping strategies must be employed to move past IS. These strategies involve proactive and intentional steps to address the negative thought patterns associated with IS. It is important to recognize that not all negative thoughts are unrealistic or distorted, and similarly, not all unrealistic thoughts are negative [29]. If an impostor struggles significantly with anxiety and depression and seeks to better understand this discomfort, applying for psychoanalysis is one approach. It is observed that an impostor sufferer employs splitting as a defence mechanism in their object world, and later transitioning to another form of splitting [28]. The split occurrs along the familiar lines of pleasure and displeasure. In the imposturous configuration, this split predominates, with the central dynamic involving the use of a pleasurable, grandiose fantasy to ward off painful and undervalued imagery (ibid.). IS shares features with post-traumatic stress disorder including patterns of distorted thinking that trigger maladaptive beliefs and behaviours [29].

Managing IS should involve the ability to discern between fact and fiction by means of critical observation that intervenes in own beliefs and feelings. Cognitive behavioural therapy (CBT) works by encouraging a person to see the self and the world in a more positive, realistic and useful way. Therapists can help replace negative core beliefs and critical self-talk with a more constructive, rational mindset. Also, logotherapy (LT) is a good choice comprising paradoxical intention, de-reflection and Socratic dialogues. A previous study [31] claimed that the first step to overcome IS happens by accepting it and normalizing it as a common part of learning. Recognizing and celebrating progress along the way rather than focusing on minor deficiencies is a way to conquer it. Shifting focus in the inner narrative from “all my colleagues are more talented than me” to “I am going to learn so much from my gifted colleagues” can help avert IS and its consequences. Another technique to overcome IS consists of recalling that ‘’I am here for a reason and I fully deserve it’’ (*ibid*). Consequently, the key to reducing IS lies in training the ability to discern facts from fiction and thereby weakening distorted thoughts that fuel feelings of being an imposter.

### Implications of the study

Surgeons are human beings with varying levels of ambition and mental vulnerabilities rooted in distorted inner narratives formed by early family dynamics and surrounding influences. Those exposed to heightened expectations and narcissistic demands may internalise a sense of never being good enough, constantly striving for an elusive standard of acceptance. IS serves as a mental defence mechanism, helping individuals endure disappointments and to transform setbacks into achievements. Some find strategies to manage IS effectively, while others notice its intensity diminishes with age. The demanding training and inherently stressful nature of the surgical profession make surgeons particularly vulnerable to mental health challenges. While surgery is an exhilarating yet exacting field, it is sometimes said that true mastery comes through hard-earned experience, often born of profound challenges.

### Limitations of the study

A limitation of this study was the potential for nonresponse bias due to the use of online survey methods. In some studies, the survey was distributed widely on the internet, rendering no tracking of the total number of individuals contacted or of the overall response rate, possible. In addition, those individuals who received an invitation to participate in the study and chose to complete the survey, were maybe those who suffered from IS. In other words, selection bias for the topic of interest may have occurred and exclusion criteria might have limited the generalizability of the findings.

## Conclusion

Imposter syndrome occurs with a high prevalence among surgeons. Surgeons with IS are predisposed to experience mental or physical challenges. They respond to failure with feelings of shame and humiliation. In contrast, they attribute success to external factors and create reasons for why they do not deserve praise or credit. Female surgeons experience IS more frequently than their male counterparts. Feelings of IS can naturally decline with increasing age. Risks for IS and multiple preventative measures were explored. The key to reducing IS is to train oneself to discern fact from fiction, thereby weakening the distorted thoughts that fuel feelings of being an imposter.

## Data Availability

No datasets were generated or analysed during the current study.
